# Effect of Exercises Performed with External Focus Instructions on Transversus Abdominis Thickness in Postpartum Women with Diastasis Recti

**DOI:** 10.3390/healthcare14070907

**Published:** 2026-03-31

**Authors:** Sara Cañamero de León, Tomás Bonino Covas, Mercedes Soto Gonzalez, Iria Da Cuña Carrera, Augusto Gil Pascoal

**Affiliations:** 1 Faculty of Physical Therapy, Department of Functional Biology and Health Science, University of Vigo, 36005 Pontevedra, Spain; m.soto@uvigo.gal (M.S.G.); iriadc@uvigo.gal (I.D.C.C.); 2 Escuela Internacional de Terapia Física (ESITEF), 28045 Madrid, Spain; tomasbonino@gmail.com; 3 Clinical Physiotherapy Group, Galicia Sur Health Research Institute, 36312 Vigo, Spain; 4 Biomechanics and Functional Morphology Laboratory, Interdisciplinary Centre for the Study of Human Performance, Faculty of Human Kinetics, University of Lisbon, 1499-002 Lisbon, Portugal; gpascoal@fmh.ulisboa.pt

**Keywords:** diastasis recti abdominis, transversus abdominis, postpartum period, exercise therapy, abdominal muscles, external focus, lumbopelvic stability

## Abstract

**Highlights:**

**What are the main findings?**

- Exercises performed with external focus instructions were associated with increased transversus abdominis thickness during task execution in postpartum women with diastasis recti abdominis.
- Most participants showed bilateral increases in transversus abdominis thickness during exercise execution without explicit instructions to voluntarily contract the muscle.

**What are the implications of the main findings?**

- Exercises incorporating external focus instructions may represent a promising alternative strategy for facilitating abdominal muscle recruitment during rehabilitation tasks.
- Task-oriented exercises that resemble functional activities may support the integration of abdominal muscle function into postpartum rehabilitation programs, although their impact on functional outcomes was not assessed in this study.
- External focus-based exercises may represent a potentially lower cognitive-demand approach compared to traditional core rehabilitation, as suggested by the motor learning literature.

**Abstract:**

**Background**: Diastasis recti abdominis (DRA) is a common condition in postpartum period and may affect abdominal function and trunk stability. Rehabilitation programs often include exercises targeting deep abdominal muscles such as the transversus abdominis. Instructional strategies derived from motor learning research, including the use of external focus, may influence how these exercises are performed. **Objectives**: To investigate changes in transversus abdominis muscle thickness during the execution of rehabilitation exercises performed with external focus instructions in women with DRA. **Methods**: A cross-sectional study with a within-subject quasi-experimental component was conducted in women with postpartum DRA. Ultrasound imaging was used to measure transversus abdominis thickness at rest and during the execution of therapeutic exercises performed under therapist guidance using external focus instructions. **Results**: Participants demonstrated increases in transversus abdominis thickness during exercise execution compared with resting conditions, suggesting effective abdominal muscle contraction during the tasks performed. **Conclusions**: Exercises performed with external focus instructions were associated with increases in transversus abdominis thickness during exercise execution in women with DRA. While these findings suggest that such instructional strategies may facilitate muscle recruitment during rehabilitation exercises, further controlled studies are required to compare different instructional approaches and to investigate potential motor learning effects.

## 1. Introduction

Diastasis recti abdominis (DRA) is a common musculoskeletal dysfunction in women during the postpartum period, characterized by a non-physiological separation of the rectus abdominis muscles along the linea alba. This condition can alter core stability and generate symptoms such as abdominal weakness, lower back pain, pelvic floor dysfunction, and limitations in functional mobility [1,2], negatively affecting the quality of life of the affected women.

Recently, greater emphasis has been placed on the impact of DRA on core biomechanics and its relationship with lumbopelvic stability. Studies have indicated that DRA can compromise motor control efficiency, increasing the load on compensatory structures and predispose individuals to chronic musculoskeletal problems [3,4]. Additionally, a strong correlation has been identified between DRA and pelvic floor dysfunctions, suggesting that a comprehensive rehabilitation approach is essential to improve functionality in postpartum women [3,5].

The activation of the Transversus Abdominis (TrA) is the main pillar of most DRA rehabilitation protocols [6,7,8,9] due to its stabilizing function of the Abdomino-Lumbo-Pelvic Complex (ALPC). Its activation improves the co-contraction of the pelvic floor muscles, the synergy with the diaphragm, and reduces lumbar overload [10,11]. Additionally, it provides tension to the area between both rectus abdominis muscles and decreases the distortion of the linea alba [12], favoring load transmission and force transfer.

In this context, it is important to explore intervention strategies that optimize core rehabilitation and minimize the functional limitations associated with this condition [2,13,14].

Traditional rehabilitation methods, commonly used until now, have prioritized strategies based on voluntary activation of the TrA through direct and explicit instructions with an internal focus. Techniques such as abdominal hollowing have been used with the aim of reducing the inter-rectus distance and strengthening the deep core muscles [15,16,17]. However, these strategies require conscious control of muscle activation, which can create barriers to movement automation and limit the transfer of skills to daily functional activities [18,19].

Therapeutic exercise in the current management of diastasis can contribute to the reduction in muscle separation, but its effectiveness is limited in some cases. It can be complemented with a more comprehensive approach that includes specific morphological evaluation of each patient [20] and a combination of therapeutic strategies adapted to their needs and that can be extrapolated to daily life tasks [21,22].

In recent years, instructional strategies derived from motor learning research, including the use of external focus, have been explored as alternative approaches in rehabilitation. Unlike traditional strategies that emphasize conscious control of specific muscles, external focus directs attention toward the effects of movement in the environment rather than toward the body segments involved in the task. According to the motor learning literature, external focus instructions have been associated with more automatic movement control and more efficient task execution in different motor tasks [23,24].

Strategies based on external focus have shown promising results in optimizing motor performance in rehabilitation contexts [24]. Directing the patient’s attention toward the outcome of the movement, rather than toward specific muscle contraction, may facilitate the execution of therapeutic exercises. For example, instructions such as “press the object (table, ball, chair, etc.) in front of you with your hands”, may encourage abdominal muscle recruitment without explicitly instructing the patient to voluntarily contract the transversus abdominis [25,26,27].

Despite the growing interest in attentional focus strategies in rehabilitation, their application in women with DRA remains limited. Exploring how exercises performed with external focus instructions influence abdominal muscle contraction may provide useful insights for postpartum rehabilitation.

Therefore, the aim of this study was to evaluate immediate changes in transversus abdominis muscle thickness during the execution of six therapeutic exercises performed with external focus instructions in women with diastasis recti abdominis. The study focused on the immediate response observed during exercise execution.

## 2. Materials and Methods

### 2.1. Study Design

A cross-sectional observational study with a within-subject quasi-experimental component was conducted to evaluate the immediate effect of rehabilitation exercises performed with external focus instructions on transverse abdominal muscle thickness. During the experimental session, participants performed a series of therapist-guided exercises, and muscle thickness was compared between resting and contraction conditions.

Ethical approval was granted by the Research Ethics Committee of the Fundación Jiménez Díaz (11 June 2024, record no. 11/24). The study was conducted in accordance with the principles of the Declaration of Helsinki.

### 2.2. Participants

Women in the postpartum period between 6 and 12 weeks after delivery, were included in this study. They were referred to by the primary care service of the Community of Madrid. To be eligible, participants needed to have an inter-rectus distance greater than 2 cm [1,28,29,30] at any of the three points measured on the linea alba at rest. Exclusion criteria were pregnancy and presence of neurological or autoimmune tissue disorders.

Sample size estimation was conducted using G*Power 3.1.9.2 for a paired *t*-test, considering a two-tailed analysis with a significance level of 0.05, statistical power of 0.80, a parameter (δ) of 3.08, a critical t-value of 2.13, and 15 degrees of freedom. The effect size (Cohen’s dz) was estimated at 0.77. The effect size calculation was based on data published by Hides et al. (2007) [31], which reported mean transversus abdominis (TrA) thicknesses of 3.5 mm (SD = 0.8 mm) at rest and 4.8 mm (SD = 1.3 mm) during contraction, corresponding to a Cohen’s dz of 1.145. To adopt a more conservative approach, we assumed a smaller difference of 1 mm in TrA thickness between rest and contraction, whereas Hides et al. (2007) [31] reported a difference of 1.3 mm. Additionally, we accounted for greater variability in data distribution by using a standard deviation (SD) of 1.3 mm, instead of the 0.8 mm SD reported in the reference study. These methodological adjustments provide a more cautious estimation of the required sample size, reducing the risk of underestimating data variability in our target population.

The analysis indicated that a minimum of 16 participants would be required to detect a statistically significant difference in TrA thickness between rest and contraction. To ensure robustness and account for potential dropouts, the final sample size was set at 18 participants.

### 2.3. Measurements

The parameters analyzed in this study were the inter-rectus distance (IRD) and the transversus abdominis (TrA) muscle thickness, both measured using ultrasound imaging. Ultrasound images were acquired with minimal pressure applied to the probe, using a thick layer of gel to ensure optimal coupling between the skin and the transducer. The ultrasound system used was the Consona N8 (Mindray, Shenzhen, China), equipped with a 5–13 MHz linear transducer in B-mode, and trapezoidal and/or panoramic images were selected when necessary. All measurements were performed by a trained healthcare professional with over five years of experience in musculoskeletal ultrasound and abdominal wall evaluation.

For IRD measurements, the areas of measurement were marked to ensure consistent transducer positioning. A soluble marker was used to identify three specific points along the linea alba: 5 cm above the navel (upper point), at the upper edge of the umbilicus (middle point), and 2 cm below the umbilicus (lower point) [2,30,31,32,33,34,35]. Participants were positioned supine at rest, with their legs and arms extended alongside their body. Images were captured at the end of a relaxed exhalation, as recommended by previous studies [36], with the fascia of each rectus abdominis muscle belly serving as the reference point for image acquisition. IRD measurements were performed using the digital caliper embedded in the ultrasound system, with the medial edges of both rectus abdominis muscles serving as reference points for the width of the linea alba.

For the assessment of TrA muscle thickness, participants were positioned supine with their legs and arms extended at their sides. Measurements were taken bilaterally on both the right and left sides under two conditions: (1) at rest, at the end of a relaxed exhalation (before initiating the exercise in the starting position), and (2) during the execution of each of the six exercises (Table 1), with the image captured at the point of maximum TrA thickness. The transducer was placed transversely on the anterolateral side of the abdomen, midway between the anterior superior iliac spine and the lower edge of the rib cage. This positioning ensured that the medial edge of the TrA was visualized in a “V” shape for proper muscle evaluation. To measure muscle thickness, a reference point was located 10 mm from the edge of the TrA, and a perpendicular line was drawn to the hyperechoic superior and inferior fascial boundaries [12,36,37].

### 2.4. Exercise Protocol

Six exercises performed with external focus instructions were evaluated (Table 1), comparing transverse abdominis (TrA) thickness at rest and during exercise execution. The order of exercises was randomized using a simple randomization method.

Before each task, the therapist demonstrated the starting position and provided verbal instructions to ensure correct execution. Each exercise was performed once during the experimental session and maintained for approximately 5 s, during which ultrasound images were acquired at the point of maximum TrA thickness.

All exercises were performed under therapist supervision and required varying levels of postural stabilization and interaction with external objects. A 5-min rest period was provided between exercises to minimize fatigue and reduce the risk of execution errors.

The tasks included unilateral support and force transmission through the upper or lower limbs, which required lumbopelvic stabilization during task execution. These biomechanical characteristics were expected to require recruitment of the abdominal musculature to maintain trunk stability.

### 2.5. Statistical Analysis

The normal distribution of the dependent variable, transversus abdominis (TrA) muscle thickness, measured both under contraction and at rest, was assessed using the Shapiro–Wilk test. Upon confirming the normality of the data, paired sample *t*-tests were performed to compare TrA thickness measurements between contraction and rest within the same group of women. All statistical analyses were carried out using SPSS software, version 24 (SPSS Inc., Chicago, IL, USA), with a significance level set at 5% (α = 0.05) for all tests. The results were interpreted with respect to the defined significance level to assess the differences in TrA thickness under the two conditions.

## 3. Results

Eighteen postpartum women with diastasis rectus abdominis participated in this study. Table 2 shows the demographic data of the sample.

The results of the study are presented in Table 2 and Table 3, which display the mean values, standard deviations (SD), differences, *p*-values, 95% confidence intervals (CI), and Cohen’s d for each exercise performed under resting and contraction conditions, respectively, for the right and left sides of the transverse abdominis (TrA).

The results for the right side of the TrA (Table 3). Significant differences between the resting and contraction conditions were found for most exercises. Effect sizes, as indicated by Cohen’s d, ranged from small to large, with Exercise 6 showing the largest effect size (Cohen’s d = 1.46). The differences in TrA thickness were consistent, with greater muscle thickness during the contraction condition compared to rest. Notably, Exercises 1, 3, 4, and 5 displayed moderate to large effect sizes (Cohen’s d ≥ 0.43), while Exercise 2 exhibited a small effect (Cohen’s d = 0.53).

Like the right side, significant differences were observed between resting and contraction conditions (Table 3). The largest effect sizes were observed in Exercises 5 and 6 (Cohen’s d = 2.51 and 2.41, respectively), indicating a very strong effect of contraction on TrA thickness. Effect sizes for Exercises 1 to 4 ranged from moderate to large (Cohen’s d ≥ 0.73), with Exercise 2 having a small effect size (Cohen’s d = 0.33). Overall, the results suggest greater TrA thickness during contraction, with significant differences across both sides.

In conclusion, the contraction condition consistently led to increased Transversus Abdominis thickness compared to the resting condition, with varying effect sizes depending on the exercise and side. The left side generally showed larger effect sizes than the right side, particularly in Exercises 5 and 6.

## 4. Discussion

This study explored the use of rehabilitation exercises performed with external focus instructions in women with diastasis recti abdominis (DRA), examining changes in transversus abdominis (TrA) thickness during task execution. The results indicate that participants were able to produce measurable increases in TrA thickness while performing the exercises, suggesting that the exercises were capable of eliciting abdominal muscle recruitment during task execution. These responses should be interpreted as immediate performance effects observed during exercise execution.

The exercises were designed using verbal cues that directed attention toward the effects of the movement rather than toward specific body segments. According to the motor learning literature, this type of attentional focus has been associated with more efficient movement execution and more automatic control strategies in motor learning [22,24,38,39,40,41,42]. In rehabilitation settings, such approaches may help patients perform therapeutic tasks in a manner that more closely resembles functional movements, although this was not directly assessed in the present study. In the postpartum period, adequate recruitment of deep abdominal muscles is particularly relevant, as these muscles contribute to stabilization of the abdomino-lumbo-pelvic complex and support postural control, coordination, and efficient limb movement [10,11,38,39].

The results obtained across the different exercises suggest that tasks involving postural stabilization and interaction with external elements may encourage abdominal muscle recruitment during movement execution. Most exercises produced significant increases in TrA thickness, while exercise 2 showed a tendency toward thickening without reaching statistical significance. One possible explanation may be related to the greater postural demands of the starting position, which may have required higher baseline muscle activity. Previous research has highlighted the role of the TrA in maintaining postural stability during tasks involving unilateral support and balance control [43]. Similarly, Tsartsapakis et al. [44] reported that movements involving trunk or limb displacement may require greater TrA recruitment to maintain spinal stability compared with exercises performed in more neutral positions.

Although increases in TrA thickness were observed bilaterally, slightly greater responses were identified on the left side. This asymmetry may be associated with unilateral support requirements or with the side responsible for resisting movement during the exercises. Previous studies have shown that exercises involving unilateral support can generate greater contralateral muscle thickness responses [45], and lateral dominance has also been suggested as a factor influencing abdominal muscle activation patterns during stabilization tasks [27].

It is important to interpret these findings with methodological caution. Ultrasound imaging allows the observation of morphological changes in muscle thickness during contraction; however, these measurements represent an indirect indicator of muscle contraction and do not necessarily reflect selective activation of the TrA or improvements in motor control. Additional methods, such as electromyography or more comprehensive functional assessments, would be required to better understand neuromuscular coordination.

Furthermore, the present study evaluated only immediate motor performance during exercise execution, rather than motor learning. Motor learning typically requires the assessment of retention, transfer, or performance under dual-task conditions. As these outcomes were not examined in this study, no conclusions regarding learning processes can be established.

Previous studies investigating TrA activation in women with DRA have largely focused on explicit instruction strategies and internal attentional focus, particularly through the abdominal hollowing maneuver [15]. A survey conducted by Keeler et al. [46] reported that physiotherapists frequently use exercises aimed at strengthening and activating the TrA, often combined with pelvic floor training. The present approach differs by incorporating external focus cues and functional tasks, which may offer an alternative way of facilitating abdominal muscle recruitment during rehabilitation exercises.

From a clinical perspective, exercises that involve interaction with external elements and resemble functional activities may help integrate abdominal muscle recruitment into movement tasks relevant to daily life, particularly considering differences in abdominopelvic function related to parity [47]. Such strategies may support the incorporation of such exercises into postpartum rehabilitation programs and potentially facilitate patient engagement in therapeutic activities.

Several limitations should be considered when interpreting the findings of this study. First, the absence of a comparison condition using internal focus or traditional instruction approaches prevents determining whether the observed responses were specifically related to external focus strategies or simply reflect the effect of performing the exercises. Second, the study examined the response to a single session, and therefore it is not possible to determine whether these effects are maintained over time. Third, ultrasound measurements were performed during static task execution, which limits the analysis of muscle behavior during dynamic movement.

Future research should include controlled experimental designs comparing different instructional strategies and should investigate potential learning effects through retention and transfer assessments. Longitudinal studies may also help clarify the potential role of these approaches in influencing inter-rectus distance and functional recovery in women with DRA. Additionally, future work could explore the effects of these strategies on other outcomes relevant to rehabilitation, such as postural stability, trunk control, and coordination of the core musculature.

### 4.1. Study Limitations

This study presents some limitations that should be considered:

1. Absence of a control group: The effectiveness of external focus exercises was not compared with traditional internal focus strategies. Including a control group in future studies would allow for better evaluation of the differences between both methodologies.
2. Immediate effect: The study focused on a single training session, so it cannot be determined if the observed effects are maintained long-term. It would be important to design studies with prolonged follow-ups to evaluate the stability of changes in TrA activation.
3. Limitations in ultrasound measurement: Due to the static nature of the ultrasound used, TrA activation in motion could not be evaluated. The development of wireless equipment could allow for a more dynamic analysis in future studies.
4. Possible influence of the clinical environment: All participants were evaluated in a healthcare center, which could have influenced their response to the training. Including evaluations in habitual environments could improve the ecological validity of the results.

### 4.2. Proposals for Future Research

The findings of this study open new lines of research in the field of DRA rehabilitation:

- Longitudinal studies: Evaluate the impact of implicit learning and external focus on TrA activation over time and its relationship with the reduction in interrectal distance.
- Comparison with traditional methodologies: Include a control group that performs exercises with internal focus to determine the relative superiority of each approach.
- Analysis of other biomechanical variables: Include measurements of postural stability, motor control, and activation of other core muscles.
- Exploration of new populations: Evaluate the effectiveness of these strategies in women with severe DRA, patients with pelvic floor dysfunctions, and populations with a history of abdominal surgery.

## 5. Conclusions

Exercises performed with external focus instructions were associated with increases in transversus abdominis thickness during task execution in women with diastasis recti abdominis. These findings suggest that such exercises may facilitate abdominal muscle recruitment during task execution; however, this interpretation should be limited to immediate performance effects observed under the study conditions. Further controlled and longitudinal studies are required to determine whether these effects extend to motor learning processes, inter-rectus distance, and functional recovery.

## Figures and Tables

**Table 1:** Description of the six therapeutic exercises performed during the experimental session. The table presents the starting position, external focus instructions, biomechanical demands, and execution rules for each task. Each exercise was maintained for approximately 5 s, during which the ultrasound image of the transversus abdominis was acquired.

Exercise	Operational Description	Task/External Focus Instruction	Trunk Stabilization Requirement	Task Constraints
E1 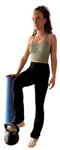	Participant stands in single-leg support with the contralateral foot placed on a weight resting on the floor.	“Lift the weight (8 kg) off the floor and hold the position until I tell you” (ultrasound image acquired during the hold phase).	Unilateral support requiring lumbopelvic stabilization and force transfer through the trunk	Do not flex the supporting knee. A foam roller may be used for light balance support.
E2 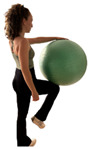	Participant stands with the ball positioned between the hand and the ipsilateral knee.	“Press the ball between your hand and knee as hard as you can and hold the pressure” (ultrasound image acquired during the hold phase).	Cross-body force transmission requiring trunk stabilization	Do not flex the supporting knee or the elbow contacting the ball.
E3 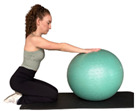	Participant stands with the ball placed between the hand and the floor.	“Press the ball against the floor as hard as you can and hold the pressure” (ultrasound image acquired during the hold phase).	Isometric force production through the upper limb requiring trunk stabilization	Do not lift the hips or bend your elbows.
E4 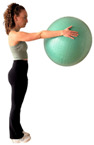	Participant stands upright facing a wall, holding a ball between one hand and the wall at approximately shoulder height with the arm extended.	“Press the ball against the wall and hold it” (while taking the ultrasound image).	Upper-limb force transmission requiring trunk stabilization	Do not move your feet or bend your elbow.
E5 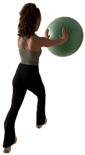	Participant stands facing a wall with the ball positioned between the hand and the wall.	“Press the ball against the wall and hold the pressure.” (ultrasound image acquired during the hold phase).	Anterior force production requiring stabilization of the trunk	Do not place the heel of the rear foot on the floor and avoid elbow flexion.
E6 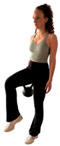	Participant stands in single-leg support holding a weight on the ipsilateral hand.	“Maintain this position until I tell you” (ultrasound image acquired during the hold phase).	Static trunk stabilization and postural control	Do not tilt the trunk. Maintain upright posture.

**Table 2:** Demographic and clinical characteristics of the participants (n = 18). Continuous variables are presented as mean and standard deviation (SD), and categorical variables as number of participants and percentage.

Value	Mean (SD/%)
AGE, years [mean (SD)]	37.2 (3.3)
POSTPARTUM WEEK [mean (SD)]	6.9 (1.0)
INTER-RECTUS DISTANCE, cm [mean (SD)]	
Umbilicus level	4.8 (2.2)
Above umbilicus	3.5 (1.5)
Below umbilicus	3.6 (1.6)
DOMINANCE [n, (%)]	Right: 16 (88.9%); Left: 2 (11.1%)
PARITY [n, (%)]	Primiparous: 5 (27.8%); Multiparous: 13 (72.0%)
DELIVERY TYPE [n, (%)]	Vaginal: 11 (61.1%); Caesarean: 4 (22.2%); Vaginal + Caesarean: 3 (16.7%)

**Table 3:** Results of transversus abdominis.

Exercise	Side	Condition	Mean (SD)	Difference			
				Mean (SD)	*p* Value	95% CI	Cohen’s d
Exercise 1	Right	Rest	3.37 (0.81)	1.06 (1.57)	0.011	[−1.84, −0.27]	0.43
		Contraction	4.43 (1.93)				
	Left	Rest	3.59 (0.88)	1.39 (1.38)	0.001	[−2.08, −0.71]	0.73
		Contraction	4.98 (1.63)				
Exercise 2	Right	Rest	4.03 (1.54)	0.46 (0.92)	0.052	[−0.91, 0.004]	0.53
		Contraction	4.49 (1.68)				
	Left	Rest	3.78 (0.84)	0.71 (1.47)	0.058	[−1.44, 0.03]	0.33
		Contraction	4.49 (1.84)				
Exercise 3	Right	Rest	3.61 (1.11)	0.99 (1.46)	0.01	[−1.72, −0.27]	0.46
		Contraction	4.60 (1.42)				
	Left	Rest	3.62 (0.94)	1.22 (1.23)	0.001	[−1.83, −0.61]	0.81
		Contraction	4.84 (1.50)				
Exercise 4	Right	Rest	3.53 (0.94)	1.06 (1.37)	0.004	[−1.74, −0.38]	0.56
		Contraction	4.59 (1.45)				
	Left	Rest	3.47 (0.82)	1.11 (1.10)	0.001	[−1.65, −0.56]	0.91
		Contraction	4.58 (1.37)				
Exercise 5	Right	Rest	3.45 (1.15)	1.24 (1.56)	0.004	[−2.01, −0.46]	0.51
		Contraction	4.69 (1.29)				
	Left	Rest	3.87 (1.35)	1.51 (0.77)	0.000	[−1.89, −1.12]	2.51
		Contraction	5.38 (1.78)				
Exercise 6	Right	Rest	3.90 (1.04)	0.63 (0.65)	0.001	[−0.95, −0.30]	1.46
		Contraction	4.52 (0.96)				
	Left	Rest	3.70 (0.93)	0.93 (0.62)	0.000	[−1.24, −0.62]	2.41
		Contraction	4.63 (1.22)				

SD: Standard Deviation; CI: Confidence interval.

## Data Availability

The data presented in this study are available from the corresponding author upon reasonable request. The data is not publicly available due to ethical and privacy restrictions.

## Abbreviations

The following abbreviations are used in this manuscript:

DRA	Diastasis Recti Abdominis
TrA	Transversus Abdominis
IRD	Inter-Rectus Distance
ALPC	Abdomino-Lumbo-Pelvic Complex
SD	Standard Deviation
CI	Confidence Interval
